# Inflammation in Preeclampsia: Genetic Biomarkers, Mechanisms, and Therapeutic Strategies

**DOI:** 10.3389/fimmu.2022.883404

**Published:** 2022-07-08

**Authors:** Yue Wang, Baoxuan Li, Yan Zhao

**Affiliations:** Department of Obstetrics and Gynaecology, Shengjing Hospital of China Medical University, Shenyang, China

**Keywords:** preeclampsia, inflammation, machine learning, regulatory pathways, curcumin

## Abstract

**Objective:**

Preeclampsia is a common and serious complication of pregnancy, posing a threat to maternal and fetal safety due to the lack of effective biomarkers and treatment strategies. This study aimed to identify potential biomarkers that can be used to predict preeclampsia and identify the molecular mechanisms of preeclampsia pathogenesis and drug prediction at the transcriptome level.

**Methods:**

We analyzed differential expression genes (DEGs) in preeclampsia and non-preeclampsia groups in the GSE75010 dataset, cross-linking with extracted inflammatory response-related genes to obtain differentially expressed inflammation-related genes (DINRGs). Enrichment analysis and protein-protein interaction (PPI) networks were constructed to understand the functions and enrichment pathways. Machine learning models were used to identify key genes associated with preeclampsia and build a nomogram in the training set, which was validated in the validation set. The R package RcisTarget was used to predict transcription factors, and Cytoscape was used to construct miRNA-mRNA pathways, which could identify the molecular mechanisms. Then, we conducted molecular docking of the obtained key genes *INHBA* (inhibin subunit beta A), *OPRK1* (opioid receptor kappa 1), and *TPBG* (trophoblast glycoprotein), as well as predicted transcription factors with drug molecules. Additionally, the CIBERSORT method explored the differences in immune cell infiltration between preeclampsia and non-preeclampsia samples based on the GSE75010 dataset.

**Results:**

A total of 69 DINRGs associated with preeclampsia patients were screened. *INHBA, OPRK1*, and *TPBG* were the key genes based on machine learning models. A nomogram for prediction was further constructed, and the receiver operating curves (ROCs) showed good performance. Based on the transcriptome level of key genes, we proposed that RELA-miR-548K/miR-1206-TPBG may be a potential RNA regulatory pathway regulating the progression of early preeclampsia. Molecular docking suggested the effectiveness of curcumin in the treatment of preeclampsia. Additionally, regulatory T cells (Tregs) and resting mast cells were significantly different between the two groups.

**Conclusion:**

In summary, we identified three key inflammation-associated genes, namely *INHBA, OPRK1*, and *TPBG*, which can be used as potential genetic biomarkers for preeclampsia prediction and treatment, and established a nomogram as a predictive model. Additionally, we provided insights into the mechanisms of preeclampsia development at the transcriptome level and performed corresponding drug predictions.

## Introduction

Preeclampsia is a serious complication specific to pregnancy, presenting as a syndrome of hypertension with multisystem involvement and damage that first appears after 20 weeks of gestation. The incidence of hypertension and preeclampsia in pregnancy has increased alarmingly in the last 30 years (1). With a global prevalence of 3%–5%, preeclampsia is a significant cause of maternal and fetal morbidity and mortality and poses significant healthcare costs worldwide (2). Maternal mortality is even higher when preeclampsia presents with severe manifestations or eclampsia or hemolysis, elevated liver enzymes, and low platelets (HELLP) syndrome. Although preeclampsia is one of the common complications of pregnancy, its exact etiology and pathogenesis are still not precisely understood and may be heterogeneous. The only cure currently is the termination of pregnancy; however, the subsequent risk of future cardiovascular and metabolic disease remains increased in mothers with a history of preeclampsia and their children (3). Therefore, in-depth comprehension of the pathogenesis and targeted treatment is particularly important for the management of patients with preeclampsia.

Studies have shown that women experience a physiological inflammatory response during pregnancy, which is a physiological stress response helping maintain the immune balance at the maternal-fetal interface, thus, protecting the fetus from disturbances and not affecting or triggering abnormal symptoms in pregnant women (4). When the inflammatory response is overactivated and pathological inflammation develops, it can lead to immune imbalance and vascular endothelial damage, which, in turn, promote the development of pregnancy complications related to preeclampsia (5). An increasing number of studies have found that the inflammatory status at the maternal-fetal interface throughout pregnancy is associated with disease progression in preeclampsia (6–8). Due to the dysregulation of the endogenous immune response, women with preeclampsia may exhibit an excessive inflammatory response, with a significant increase in pro-inflammatory cytokines. Additionally, women in an inflammatory overreaction state during early pregnancy can develop preeclampsia (6). Several studies have shown that insufficient or inadequate resolution of inflammation plays an important role in the development of preeclampsia (7). Suppression of the inflammatory response may alleviate the development of preeclampsia (8). It is now generally accepted that the placenta plays a crucial role in the development of preeclampsia. Invasion of trophoblast cells into the uterine wall during early pregnancy causes remodeling of the uterine spiral arteries. If there is impaired invasion or not remodeled arteries at this time, hypoxia/reperfusion develops, exacerbating reactive oxygen species (ROS) production and triggering oxidative stress in the placenta. With the triggering of oxidative stress, the synthesis of pro-inflammatory factors increases, and the inflammatory response increases, which, in turn, induces vascular endothelial dysfunction. Hypoxia/reperfusion-induced placental injury is consistent with a pathological inflammatory response, which can trigger a systemic inflammatory response and endothelial damage, contributing to the development of preeclampsia (9–12). The severity of this inflammatory overreaction is determined by genetic and environmental factors (12). Curcumin has been recently demonstrated to have a therapeutic effect on preeclampsia by several studies (13). Curcumin has been shown to provide protection against placental disorders, such as preeclampsia. Recent studies found curcumin could effectively improve pregnancy outcomes in preeclamptic mice by increasing the number of live births, fetal weight, and placental weight (14–17). There are few clinical trials on curcumin application during human pregnancy. A double-blind, randomized clinical trial found no significant difference in serum cyclooxygenase (COX)-2 and interleukin (IL)-10 levels with the use of curcumin, and the author speculated that this result might be related to the low dose of applied curcumin (100 mg/d) since curcumin can be applied up to 1 g/d during non-pregnancy (18).

In this study, we used inflammatory genes and gene expression profiles downloaded from the Gene Expression Omnibus (GEO) datasets to explore the changes and biological mechanisms of inflammatory genes at the maternal-fetal interface in preeclamptic patients. We built machine learning models based on the training set for filtering the best model to identify key genes associated with inflammation. A nomogram was constructed based on key genes as a preeclampsia prediction model, which showed good performance and was validated using the validation set. We further explored the expression and potential molecular mechanisms of key genes and applied molecular docking to explore effective drugs. Besides aspirin, curcumin was included in our study. Additionally, we investigated the potential relationship between immune cells and preeclampsia.

## Materials and Methods

### Data collection

First, the profiles dataset GSE75010 was downloaded from the Gene Expression Omnibus datasets (GEO, https://www.ncbi.nlm.nih.gov/geo/), which were measured by Affymetrix Human Gene 1.0 ST Array.GSE75010 included 80 placental samples from patients with preeclampsia(PE) and 77 placenta samples from patients without preeclampsia(non-PE). Then, The list of 200 inflammatory response-related genes were obtained from the Molecular Signatures database and shown in **Supplementary Table 1**.

### Study Population

Patients with preeclampsia were defined as those with new-onset hypertension (systolic blood pressure ≥ 140 mmHg and/or diastolic blood pressure ≥ 90 mmHg) and proteinuria (24-h proteinuria ≥ 0.3 g/24 h or ≥ 2 + by dipstick) after 20 weeks of gestation. There were 80 patients with preeclampsia in GSE75010. Patients with non-preeclampsia included eligible pregnancies and patients with chronic hypertension who had developed hypertension before 20 weeks of gestation. There were 77 non-preeclampsia patients in GSE75010, which included normotensive pregnancies (n = 53) and chronic hypertensive pregnancies (n = 24). The exclusion criteria comprised patients with diabetes (pre-existing or gestational), morbid obesity (BMI ≥ 40), sickle cell anemia, or not a singleton pregnancy. The detailed clinical data are shown **in** **Supplementary Table 2**. Placenta sampling was conducted using BioBank (http://biobank.lunenfeld.ca/), and each placental tissue was sampled four times based on four quadrants (excluding the chorionic plate) (19). All data for this study were obtained from public databases; therefore, the study did not require the institutional review board approval.

### Differential Expression Analysis

We analyze the differentially expressed genes (DEGs) between preeclampsia and non-preeclampsia from the profile of GSE75010 based on the limma R package.The threshold was selected as the adjusted *P <*0.05. Subsequently, we took the intersection of 5118 DEGs and 200 inflammatory response-related genes to obtain differentially expressed inflammatory response-related genes(DINRGs).

### Protein-Protein Interaction Network of DINRGs

The STRING database (STRING 11.0, http://string-db.org/cgi/input.pl) was used to investigate the potential protein-protein interactions (PPI) among the DINRGs (20). PPI pairs with a combined score > 0.4 were extracted. Then, Cytoscape software(https://cytoscape.org/) was used to build PPI network on the basis of these protein pairs (21).

### GO and KEGG Functional Enrichment Analysis

Gene Ontology (GO) analysis which include biological process (BP), molecular function (MF) and cellular component (CC) and Kyoto Encyclopedia of Genes and Genomes (KEGG) pathway enrichment analysis were performed to reveal the enriched pathways and biological functions of DINRGs using the R package “clusterProfiler”, and further visualized the result based on the ggplot2 package. P < 0.05 was considered as the threshold.

### Construction and Evaluation of Machine Learning-Based Models

We randomly divided all samples in the GSE75010 dataset into training and validation sets in a ratio of 7:3. We screened five important genes with |logFC| >0.4 as the criterion from the differentially expressed inflammation-related genes (DINRGs). The support vector machine (SVM) model is a binary classification model in which the basic model is a linear classifier defined on the feature space with the maximum interval, and the maximum interval makes it different from perceptron. The random forest (RF) model is an integrated learning method that performs classification or regression by integrating the voting results of multiple decision trees, thereby preventing the overfitting of the training data. The generalized linear model (GLM) is a generalization of the linear regression model, which adds other constraints to the linear model and greatly extends the standard linear model. GLM judges the model effect by observing the plot between residual and predicted values, which not only makes classification but also gives the confidence level of detection. On the basis of the caret R package and the training set, the SVM model, RF model, and GLM were respectively constructed with the expression values of DINRGs as explanatory variables and the diagnosis of preeclampsia or non-preeclampsia as the categorical responsive value. The three models were further analyzed, and visualization using the explanatory properties of the DALEX package and cumulative residual and box plot distributions were created. The residual distributions were drawn to obtain the best model. Then, penalized regression was applied to reduce the number of DINRGs. We analyzed the significance of different variables in different models, and three key genes were identified for further analysis by machine learning GLM.

### Establishment and Assessment of Nomogram

Nomograms can be used to combine multiple indicators to predict disease onset or progression. Based on the key genes, a nomogram model for predicting the occurrence of preeclampsia was formulated by the rms package. The receiver operating characteristic (ROC) analysis was applied to determine the sensitivity and precision of the genetic signature and nomogram model based on the pROC R package, and we applied the validation set to verify the genetic features. Moreover, calibration curves were applied to the evaluation of the predictive capability of the nomogram model, and we used the validation set to verify that predictive capability. Furthermore, decision curve analysis (DCA) curves for genetic signature and nomogram model were simultaneously plotted by using the ggDCA package to assess the clinical utility of the model.

### Expression and Potential Molecular Mechanisms of Key Genes

First, heatmaps were expressed based on the R package pheatmap, where different colors represent expression trends in different tissues. Then, the chromosomal locations of the most vital genes were shown. Moreover, we compared the expression of key genes between preeclampsia and non-preeclampsia groups and analyzed the genetic correlations.

Next, we investigated the specific regulatory mechanisms involved in key genes to explore the potential molecular mechanisms by which key genes influence the progression of preeclampsia. We performed a transcriptional regulation analysis of key genes. Motif, as a typical sequence or a structure, is increasingly important in gene regulation analysis. Transcription factors usually exert transcriptional regulation by recognizing motifs and binding to regulatory regions of genes. We extracted upstream motifs based on key genes, and then each motif was subjected to normalized enrichment scoring (NES) and enrichment analysis using cumulative recovery curves. Subsequently, the R package RcisTarget was used to predict transcription factors based on motifs. We used rcistarget.hg19.motifdb.cisbpont.500bp basis into the Gene-motif rankings database.

In addition, we constructed ceRNA regulatory networks based on key genes. MirRDB Online Database (http://mirdb.org/cgi-bin/search.cgi) is a commonly used website for querying mRNA-miRNA relationships. We used mirRDB online database to predict mRNA-miRNA interactions and built miRNA-mRNA network and visualized it with cytoscape.

Finally, we further investigated the relationship between obtained transcription factors and miRNA, both based on key genes. We used the TransmiR v2.0 database (http://www.cuilab.cn/transmir) to get the transcription factor (TF)-miRNA regulations based on key genes.

### Molecular Docking

We searched DrugBank (https://go.drugbank.com) for drugs targeting preeclampsia to explore the clinical potential of key genes. Three drugs that were searchable included aspirin, hydrochlorothiazide, and magnesium sulfate. Hydrochlorothiazide, which is used to treat edema associated with hypertension, and magnesium sulfate, which is used to treat convulsions and tetany during pregnancy, were not discussed for the lack of preventive and therapeutic effects. Additionally, curcumin was included in our study. The Protein Data Bank (PDB) (http://www.rcsb.org) and Pub Chem database (https://pubchem.ncbi.nlm.nih.gov) were searched to obtain the structural data of the target proteins and drug components. The protein molecules were then converted to *.pdbqt format and docked using Auto Dock1.2.0 software. Molecular docking was performed by AutoDockTools, and the folding patterns and molecular interactions between target proteins and ligand molecules were examined to derive binding energies for screening.

### Immune Infiltration Analysis

The proportion of 22 infiltrating immune cells in the samples of the GSE75010 dataset were analyzed using the CIBERSORT algorithm (https://cibersort.stanford.edu/). The top 20 classes of immune cells obtained from Cibersort’s calculations were divided into four categories: Total lymphocytes, Total dendritic cell, Total macrophage and Total mast cell,and then we further analyzed the differences in the distribution of immune cells between the preeclampsia and non-preeclampsia groups.

### Statistical Analysis

R software(version 4.0.3, http://www.r-project.org) and SPSS (version 23.0) were used for statistical analysis. P<0.05 indicates that the difference is statistically significant.

## Results

### Differentially Expression Analysis, Protein-Protein Interaction Analysis and Enrichment Analysis

**Figure 1:**
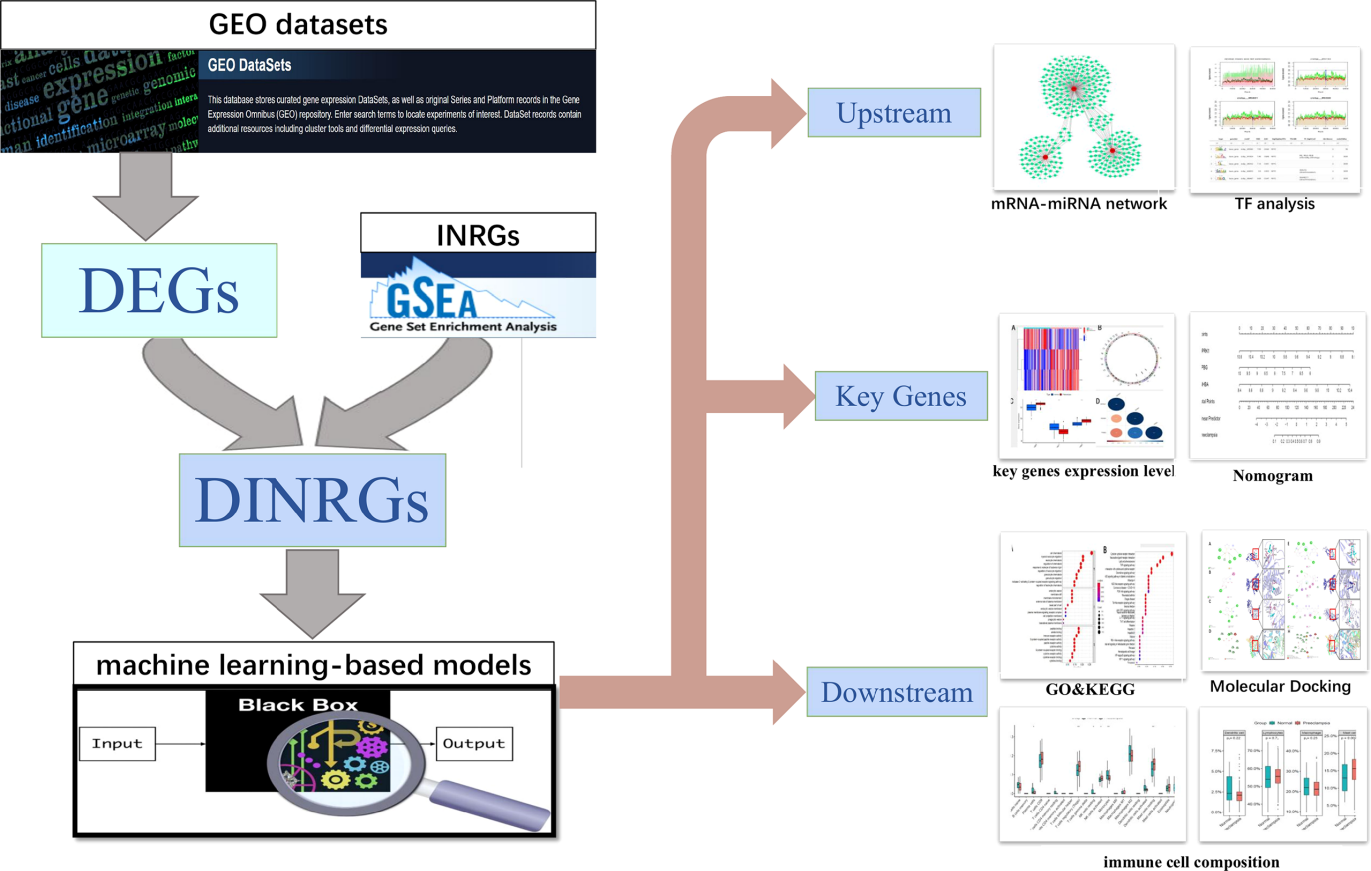
Study flowchart. GEO datasets, Gene Expression Omnibus datasets; DEGs, differentially expressed genes; INRGs, Inflammatory response-related genes; DINRGs, differentially inflammatory response-related genes; TF, transcription factors.

**Figure 2:**
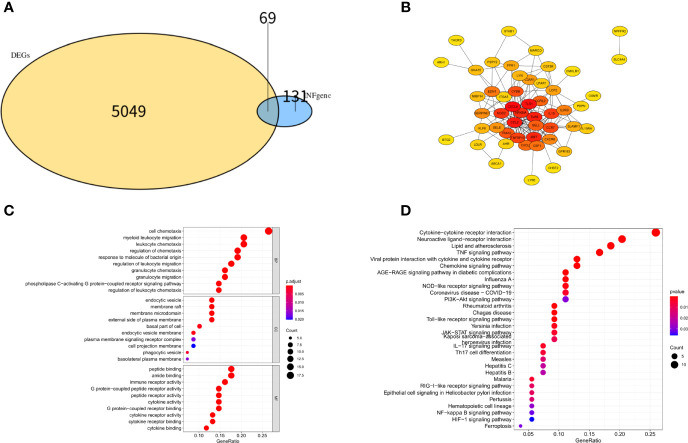
Differentially expression analysis, PPI network analysis and enrichment analysis of DINRGs. **(A)**The intersection of DEGs in GSE75010 dataset and INRGs. **(B)** The PPI network analysis for the 69 DINRGs. **(C)** Top 10 most significant GO analysis. **(D)** Top 10 most significant KEGG pathways.

The flow chart for this study is presented in **Figure 1**. Important clinical differences between the preeclampsia and non-preeclampsia groups are presented in **Table 1**. After adjusted P<0.05 as a threshold, 5118 differentially expressed genes (DEGs) were obtained between preeclampsia and non-preeclampsia samples in GSE75010.We further intersected 5118 DEGs with 200 inflammatory response-related genes, and 69 differentially expressed inflammatory response-related genes were identified (**Figure 2A**). The protein-protein interaction (PPI) network of 69 DINRGs was investigated using STRING. Then, the data were imported into Cytoscape, and the PPI network was established (**Figure 2B**). We further analyzed 69 DINRGs for functional annotation and pathway enrichment. The top 10 of Gene Ontology (GO) enrichment analysis and Kyoto Encyclopedia of Genes and Genomes (KEGG) enrichment analysis are shown as bubble charts (**Figure 2C**).GO enrichment analysis was performed to reveal the biology process (BP), molecular function (MF), and cellular components (CC) of DINRGs. The top 3 GO_BPs were cell chemotaxis, myeloid leukocyte migration, and leukocyte chemotaxis. The top 3 of GO_CCs were endocytic vesicle, membrane raft, and membrane microdomain. The top 3 of GO_MFs were peptide binding, amide binding, and immune receptor activity. The KEGG enrichment analysis revealed that cytokine-cytokine receptor interaction played an important role in preeclampsia patients. Additionally, neuroactive ligand-receptor interaction and lipid and atherosclerosis also had a significant role in the development of preeclampsia (**Figure 2D**). These results showed that placenta inflammation might play a major role in preeclampsia.

**Table 1:** Clinical Differences between the preeclampsia and non-preeclampsia groups.

Characteristic	PE (n=80)	Non-PE (n=77)	p value
Demographic data			
Maternal age, year	33.2 ± 5.9	33.2 ± 5.3	0.995
Maternal ethnicity (NA and Others excluded)			0.121
Caucasian,%	56.6 (43/76)	59.2 (42/71)	
Black,%	22.4 (17/76)	9.9 (7/71)	
Asian,%	14.5 (11/76)	25.4 (18/71)	
East Indian,%	6.6 (5/76)	5.6 (4/71)	
Previous nulliparity	61.3 (49/80)	45.5 (35/77)	0.047
Clinical features			
BMI >25 kg/m^2^	56.5 (39/69)	35.7 (25/70)	0.014
Maximum SBP, mmHg	169.8 ± 18.1	136.1 ± 23.5	<0.001
Maximum DBP, mmHg	107.3 ± 9.7	85.3 ± 14.7	<0.001
Previous hypertensive pregnancy	59.3 (16/27)	18.8 (6/32)	<0.001
Clinical data			
GA at delivery,weeks	32.3 ± 3.6	34.0 ± 4.7	0.013
Delivery <34 week,%	61.3 (49/80)	45.5 (35/77)	0.047
Placental weight z-score	-1.1 ± 0.9	-0.3 ± 1.2	<0.001
Newborn weight z-score	-1.1 ± 0.8	-0.2 ± 1.2	<0.001
Cord diameter, cm	1.2 ± 0.4	1.2 ± 0.3	0.400

SBP, Systolic Blood Pressure; DBP, Diastolic Blood Pressure; GA, Gestational Age.

### Construction and Validation of Machine Learning Models

**Figure 3:**
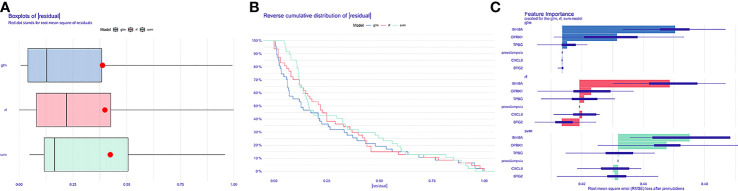
Construction and validation of machine learning-based models **(A)** Cumulative residual distribution map of different machine learning models. **(B)** Boxplots of different machine learning models.(C)The importance of explanatory variables in the different machine learning models.

Five genes (*OPRK1, BTG2, CXCL8, TPBG* and *INHBA*) with log|FC|>0.4 were selected from 69 DINRGs as key genes for constructing the machine learning models. The machine learning models were developed by the RF model, SVM model, and GLM based on the GSE75010 dataset. GSE75010 dataset was randomly divided into training and validation sets in the ratio of 7:3. The RF model, SVM model, and GLM were built using the Caret R package, and then the explanatory analysis of the three models was performed with the explain function of the DALEX package, followed by the cumulative residual distribution and the boxplot plotted based on the training set. The red dots in the boxplot represent the loss function (the root means square of the residuals), meaning that the absence of this variable brings the degree to the predicted value of the response variable. As shown in **Figures 3A, B**, GLM is considered to be the most appropriate model. Then, **Figure 3C** shows the relationship between a single continuous-type explanatory variable and the response variable. We can find that the three explanatory variables *INHBA, OPRK1*, and *TPBG* in the GLM model contributed more to the model.

### Establishment and Assessment of Nomogram

**Figure 4:**
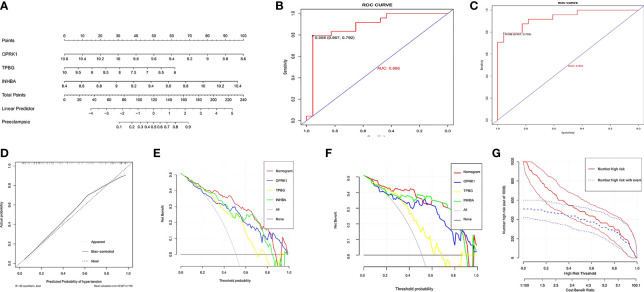
Establishment and assessment of nomogram. **(A)** Nomogram predicting the occurrence of preeclampsia. **(B)** ROC curves in the training set.(C) ROC curves in the validation set.(D) Calibration curve;(E)Decision curves in the training set;(F)Decision curves in the validation set;(G)clinical impact curve.

A nomogram was constructed based on three explanatory variables *INHBA, OPRK1*, and *TPBG* (**Figure 4A**). Each risk factor was assigned a score, and the sum of the scores of these indicators was used as the total score to predict the probability of preeclampsia in each patient. The results of the ROC curve analysis showed that the area under the curve (AUC) of the risk score in the training set could reach 0.886 (**Figure 4B**). Similarly, we applied the validation set data to plot the ROC curve, and the AUC of the risk score could reach 0.933 (**Figure 4C**). The standard curve can be used to evaluate the predictive ability of the nomogram, and the calibration curve close to 45° indicates that the model has a good predictive ability. As shown in **Figure 4D**, the level of the calibration curve in the training set overlapped well with the standard curve, indicating that the nomogram has high accuracy for predicting preeclampsia. Then, we plotted the DCA curves considering the clinical usefulness of the risk model. In **Figure 4E**, we can observe that the nomogram has a large AUC and is far from the two extreme curves, which is more valuable than other models. We also performed the validation in the validation set, and the results were consistent with the training set (**Figure 4F**). **Figure 4G** provides a more intuitive evaluation to assess the clinical application of the nomogram. In **Figure 4G**, the number high risk curve and the number high risk with event curve are close to 0.4–1 (high risk threshold region), indicating that the nomogram has extraordinary predictive power. Based on these results, it is clear that the risk score model has good predictive power, and the three key genes play a critical role in the development of preeclampsia.

### Further Analysis of *INHBA, OPRK1*, and *TPBG*

**Figure 5:**
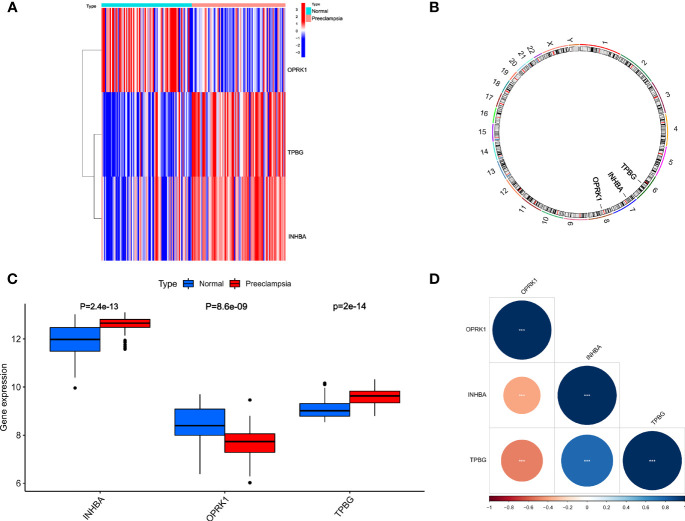
Relative expression level and correlation among *INHBA, OPRK1* and *TPBG*. **(A)** The heatmap of *INHBA, OPRK1* and *TPBG* between PE group and non-PE group. **(B)** The chromosomal locations of *INHBA, OPRK1* and *TPBG*. **(C)** The relative expression level of *INHBA, OPRK1* and *TPBG* between PE group and non-PE group. **(D)** The correlation among *INHBA, OPRK1* and *TPBG*.

We further analyzed *INHBA, OPRK1*, and *TPBG*, which played a crucial role in the development of preeclampsia. As shown in **Figure 5A**, the expression of *INHBA, OPRK1*, and *TPBG* showed significant differences between preeclampsia and non-preeclampsia samples. The positions of *INHBA, OPRK1*, and *TPBG* in the chromosomes are labeled in **Figure 5B**. Subsequently, we analyzed the expression of *INHBA, OPRK1*, and *TPBG* in preeclampsia placental tissue and non-preeclampsia placental tissue (**Figure 5C**). We found that the expression of *INHBA* and *TPBG* was higher in 80 preeclamptic placental tissues than in 77 non-preeclamptic placental tissues. However, the expression of *OPRK1* was lower in preeclamptic placental tissue. Then, we analyzed the correlation of *INHBA, OPRK1*, and *TPBG*, as shown in **Figure 5D**. We found that *INHBA* and *TPBG* were negatively correlated with *OPRK1*, and *INHBA, OPRK1*, and *TPBG* had high correlation coefficients with each other.

**Figure 6:**
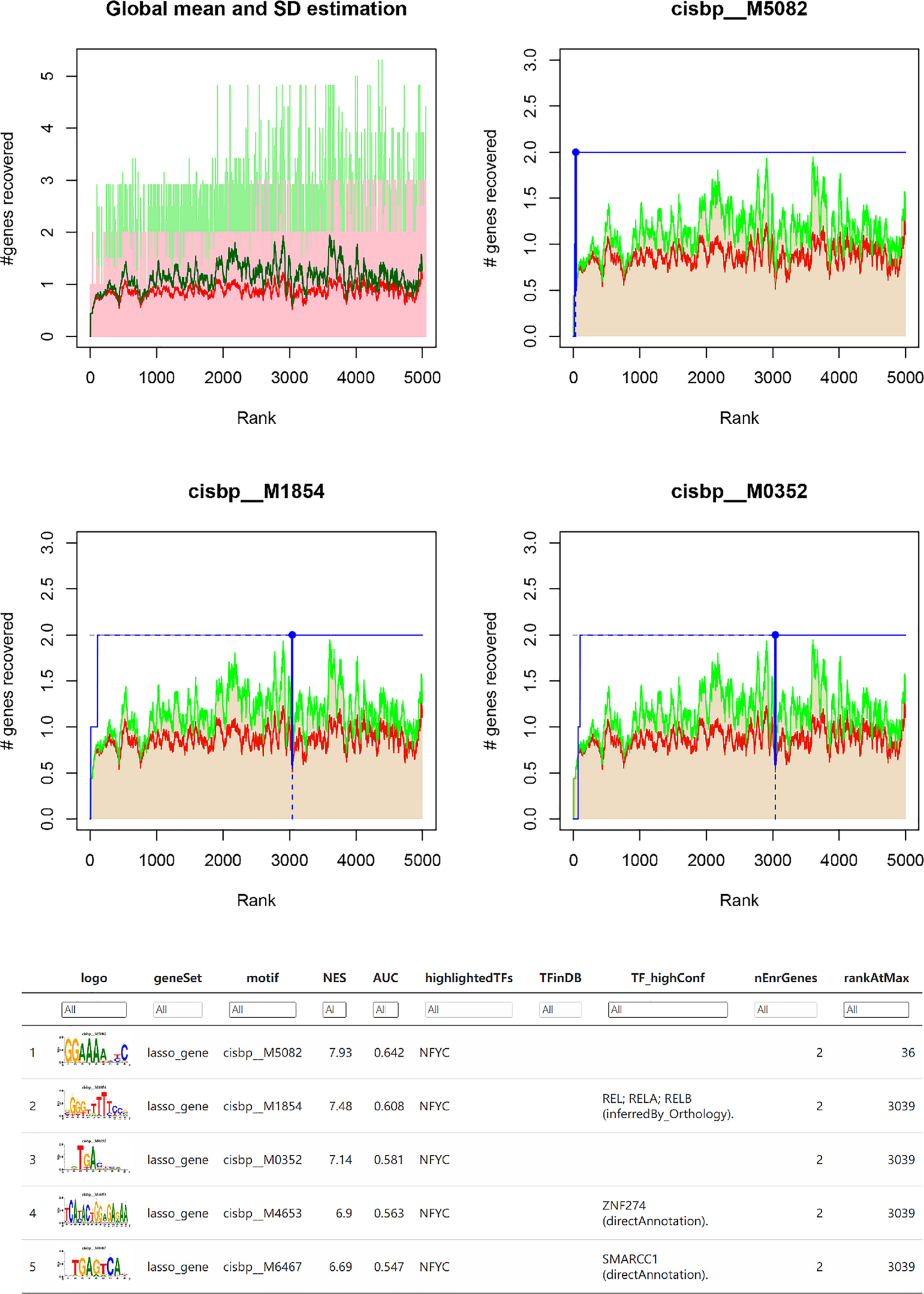
Transcriptional regulation analysis among INHBA, OPRK1 and TPBGs.

Subsequently, we investigated the specific regulatory mechanisms of the key genes *INHBA, OPRK1*, and *TPBG.* Enrichment analysis of these transcription factors was performed using cumulative recovery curves. The analysis showed that the transcription factors REL, RELA, RELB, ZNF274, and SMARCC1 were the main regulatory factors in the gene set. *INHBA* and *TPBG* were enriched in this main motif. The motifs enriched to key genes and the corresponding main transcription factors are shown in **Figure 6**.

**Figure 7:**
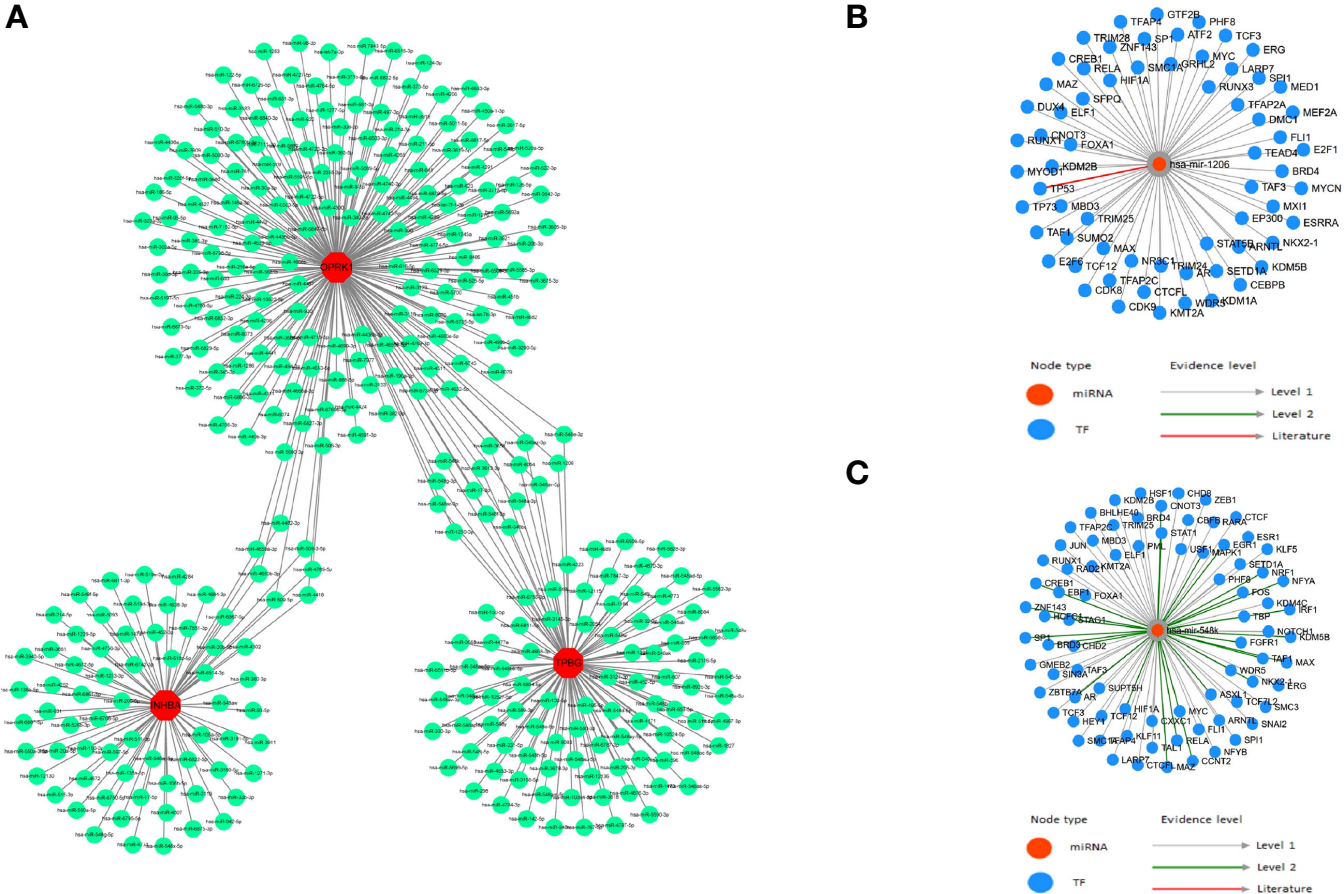
TF-miRNA regulations. **(A)** mRNA-miRNA relationship pairs; **(B)** has-miR-548k and associated TF regulation; **(C)** has-miR-1206 and associated TF regulation.

It is well known that miRNAs can induce gene silencing and down-regulate gene expression by binding mRNAs. We performed reverse prediction of three key genes through mirRDB online database which predicted 359 miRNAs with a total of 359 mRNA-miRNA relationship pairs, and successfully constructed the core gene-related mRNA-miRNA regulatory network (**Figure 7A**). In addition, we explored TF-miRNA regulations on the basis of the key genes in order to further identify the mechanisms of preeclampsia disease development at the transcriptome level. We found that RELA is associated with has-miR-548k and has-miR-1206 regulation. However, the specific mechanism still needs to be further explored (**Figures 7B, C** **)**.

### Molecular Docking

**Figure 8:**
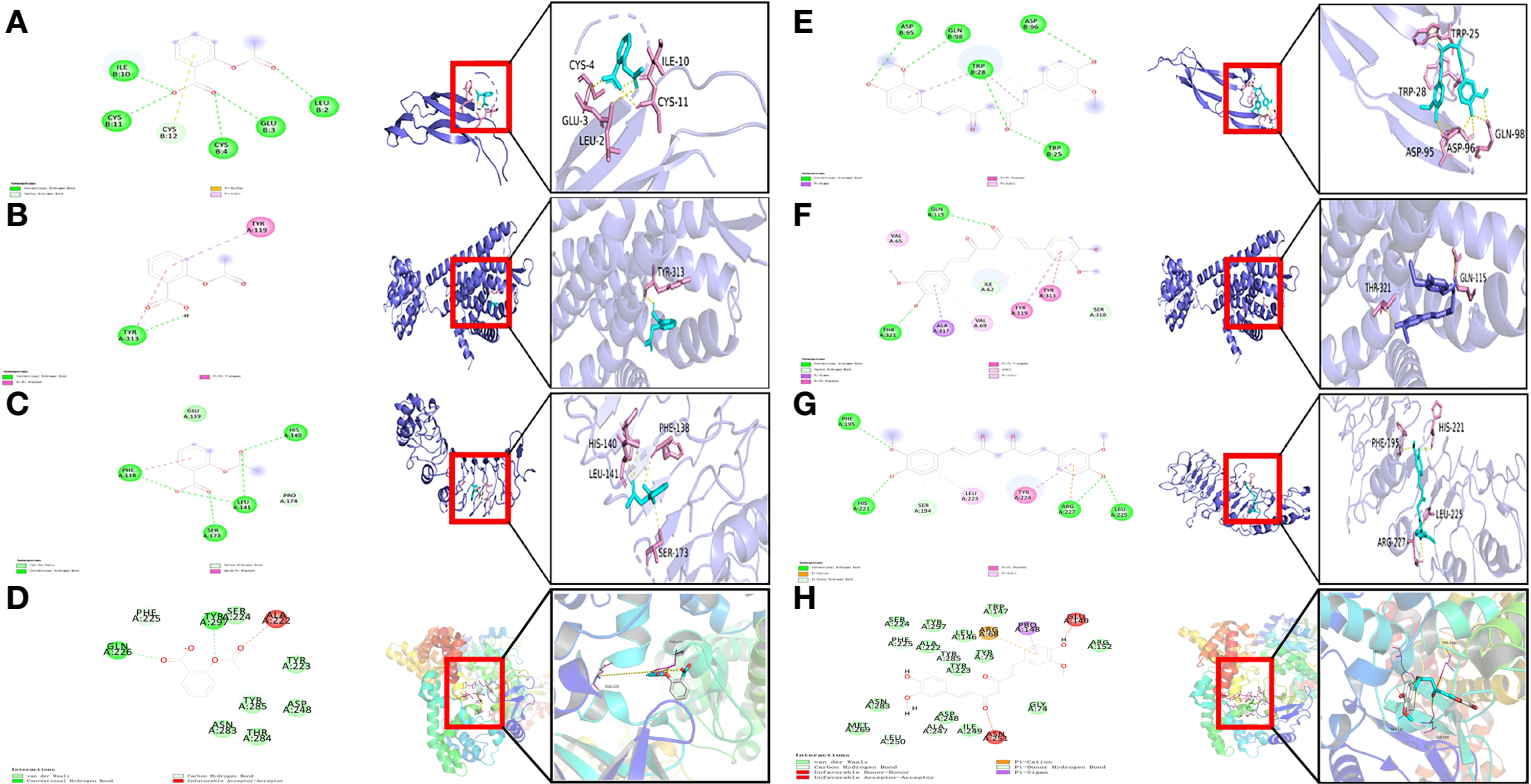
Visualization of docked poses of aspirin and curcumin with their protein target. **(A)** Binding interactions of aspirin with active site residues of INHBA-1nys; **(B)** Binding interactions of aspirin with active site residues of OPRK1-4djh; **(C)** Binding interactions of aspirin with active site residues of TPBG-4cnc; **(D)** Binding interactions of aspirin with active site residues of RELA-3qxy; **(E)** Binding interactions of curcumin with active site residues of INHBA-1nys; **(F)** Binding interactions of curcumin with active site residues of OPRK1-4djh; **(G)** Binding interactions of curcumin with active site residues of TPBG-4cnc; **(H)** Binding interactions of curcumin with active site residues of RELA-3qxy.

Three key genes and RELA were selected to act as targets for molecular docking with aspirin and curcumin, respectively. It is generally accepted that the lower the binding of the small molecule ligand to the receptor, the greater the interaction and the greater the potential activity of the component. The binding affinity that is less than -5 kcal/mol indicates spontaneous binding between the small ligand molecule and the receptor protein, while the binding affinity that is less than -7.0 kcal/mol indicates the good binding activity of the ligand to the receptor (22). According to the molecular docking results (**Table 2**), we found that the binding affinity between aspirin and TPBG-4cnc, OPRK1-4djh, and RELA-3qxy was less than −5 kcal/mol, indicating that they have certain binding activity. The binding affinity for curcumin and TPBG-4cnc, OPRK1-4djh, and RELA-3qxy was less than −7.0 kcal/mol, indicating that they have strong binding energy, which further suggests the effectiveness of curcumin in the treatment of preeclampsia. However, further studies are needed to evaluate the safety, especially the effect on the embryo in early pregnancy. Molecular docking visualization analysis was performed separately utilizing Pymol 2.2.0 software (**Figure 8**). Aspirin formed hydrogen bonds with the five amino acids CYS-4, GLU-3, LEU-2, CYS-11, and ILE-10 near the active site to INHBA-1nys; TYR-313 near the active site to OPRK1-4djh; HIS-140, LEU-141, SER-173, and PHE-138 near the active site to TPBG-4cnc; GLN-226 and TYR-297 near the active site to RELA-3qxy. Curcumin formed hydrogen bonds with five amino acids TRP-28, ASP-95, ASP-96, GLN-98, and TRP-25 near the active site to INHBA-1nys; THR-321 and GLN-115 near the active site to OPRK1-4djh; PHE-195, ARG-227, LEU-225, and HIS-221 near the active site to TPBG-4cnc; PHE-225, ALA-247, LEU-250, and TYR-285 near the active site to RELA-3qxy.

**Table 2:** The docking score of aspirin and curcumin to TPBG-4cnc\ INHBA-1nys\ OPRK1-4djh\RELA-3qxy (RMSD: 0.00).

Ligand	2D-structure	Proteins	Binding Affinity (kcal/mol)	Interacting amino acids
Aspirin		TPBG-4cnc	-5.4	HIS-140, LEU-141, SER-173, PHE-138
	C _9_ H _8_ O _4_	INHBA-1nys	-4.5	CYS-4, GLU-3, LEU-2, CYS-11, ILE-10
		OPRK1-4djh	-6	TYR-313
		RELA-3qxy	-6	GLN-226, TYR-297
Curcumin		TPBG-4cnc	-7	PHE-195, ARG-227, LEU-225, HIS-221
	C _21_ H _20_ O _6_	INHBA-1nys	-6	RP-28, ASP-95, ASP-96, GLN-98
		OPRK1-4djh	-8.5	THR-321, GLN-115
		RELA-3qxy	-8.1	PHE-225, ALA-247, LEU-250, TYR-285

### Differences in the Distribution of Immune Cells

**Figure 9:**
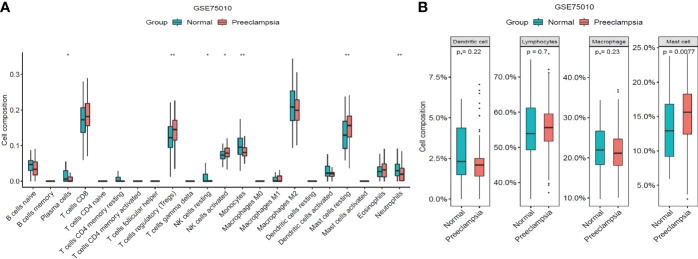
Distribution of immune cells between non-PE group and PE group. **(A)**Differences of immune cell types between non-PE group and PE group. **(B)** Differences among total lymphocytes, total dendritic cell, total macrophage and total mast cell between non-PE group and PE group. *P < 0.05, **P < 0.01.

We analyzed the proportion of 22 immune cell types infiltrating each sample in the preeclampsia and non-preeclampsia groups. There were some differences in immune cells between the preeclampsia and non-preeclampsia groups (**Figure 9A**). Regulatory T cells (Tregs), resting mast cells, monocytes, and neutrophils were significantly different between the preeclampsia and non-preeclampsia groups. To illustrate the differences in immune cells between the two groups graphically, we divided the top 20 classes of immune cells obtained from the CIBERSORT calculations into four categories: total lymphocytes, total dendritic cells, total macrophages, and total mast cells. As shown in **Figure 9B**, there is a significant difference in mast cells between the preeclampsia and non-preeclampsia groups, and mast cells are significantly higher in the preeclampsia group than in the non-preeclampsia group. The difference in immune cells between the two groups may be related to the development of preeclampsia.

## Discussion

It is currently accepted that preeclampsia can still be diagnosed even in the absence of proteinuria when accompanied by any of the following symptoms: renal insufficiency, liver involvement, neurological or hematological complications, uteroplacental dysfunction, or fetal growth restriction (23). The incidence of hypertensive disorders of pregnancy increased by 10.92%, while related mortality decreased by 30.05% in 204 countries/areas worldwide from 1990 to 2019 (24). Although maternal mortality in high-income countries has been much lower, hypertensive disorders of pregnancy can still account for 16% of maternal mortality in developed countries. In Latin America and the Caribbean, hypertensive disorders of pregnancy can be responsible for up to 26% of maternal deaths (25). It is now mostly believed that preeclampsia occurs in two stages, with placental insufficiency due to intravascular trophoblastic invasion and impaired spiral arterial remodeling in early pregnancy, followed by systemic endothelial dysfunction (26). Pathological inflammatory changes can cause local immune imbalance, affecting intravascular trophoblastic invasion and spiral artery remodeling, which, in turn, lead to placental dysfunction, representing a risk factor for the development of preeclampsia (27). Additionally, pathological inflammatory changes can lead to vascular endothelial damage, which plays an important role in the development and progression of preeclampsia (28). Although preeclampsia is a common and serious complication of pregnancy, there are no effective early prevention, diagnosis, or intervention programs that can improve maternal and newborn outcomes. Therefore, it is important to explore its possible pathogenesis and provide early prevention and timely and individualized therapy for patients with preeclampsia to improve pregnancy outcomes.

In this study, we analyzed the differential expression of inflammatory genes in gene expression profile data from preeclampsia and non-preeclampsia placental tissues, and the enrichment analysis was performed on the DEGs. Prediction models containing five DINRGs were built based on the training set by three machine learning methods, namely the RF model, GLM, and SVM model. SVM is a powerful method for building classifiers, and SVM-based classification features are widely used in genomics for discovering new biomarkers, drug targets, and driving gene understanding (29). We applied SVM to build an iterative training model, rank the influencing parameters, and then remove the lowest ranked parameters to perform feature selection and finally obtained the parameters with the maximum accuracy and lowest mean error. RF is an integrated learning method to classify or regress by combining the voting results of multiple decision trees, which is widely used in classification problems and can also be used in regression problems (30). RF uses the bootstrap resampling technique to select feature sets by random sampling and random selection, which greatly avoids the overfitting problem caused by the excessive similarity between decision trees. GLM can detect differential expression in paired designs and even specific expression changes. Given the gene-specific variability, non-linear models are faster and more reliable to fit, making the GLM more convenient and practical in genomic data (31). We integrated three different algorithms, with each having its own inherent characteristics, and found that the GLM was the best predictive model by plotting the residual distribution. Subsequently, five DINRGs were analyzed for their criticality to the GLM, and we identified *INHBA, OPRK1*, and *TPBG* as key genes.

The predictive model developed in this study consisted of three inflammatory response-related genes (*INHBA, OPRK1*, and *TPBG*). Further analyzing the three genes, we found that the expressions of *INHBA* and *TPBG* were higher in preeclamptic placental tissues than in non-preeclamptic placental tissues. Also, the expression of *OPRK1* was decreased in preeclamptic placental tissues. *INHBA*, located at 7p14.1, is a gene encoding a member of the transforming growth factor (TGF)-β protein superfamily. *INHBA* encodes subunits of activin and inhibin and has been associated with the development of preeclampsia in several studies (32–38). During early embryonic implantation, both trophoblast and ectodermal cells secrete a variety of growth factors and cytokines to regulate trophoblast differentiation and invasion at the maternal-fetal interface. It has been shown that impaired uterine spiral artery remodeling caused by insufficient trophoblast infiltration may be critical in the early stages of preeclampsia development, and upregulation of *INHBA* expression promotes human trophoblast invasion and plays an important role in early embryonic implantation (32). Activin A is a homodimeric protein encoded by *INHBA*, which can be produced by placental cells (33). Clinical data and experiments have found that activin A is elevated in preeclampsia (34–36). Studies have also shown that activator A can be elevated months before a preeclamptic seizure (37). Additionally, elevated activin A levels are strongly associated with myocardial dysfunction after preeclamptic pregnancies. It has been shown that activin A levels could predict distant cardiac impairment after preeclamptic pregnancy, implying that activin A levels may be useful for monitoring the risk of distant cerebrovascular disease (38). *OPRK1*, located at 8q11.23, encodes an opioid receptor and is a member of the family consisting of seven transmembrane G protein-coupled receptors. OPRK1 acts as a receptor for endogenous ligands and various synthetic opioid-like substances and binds to ligands to inhibit adenylate cyclase activity and neurotransmitter release. There is no information on studies directly related to *OPRK1* and preeclampsia. It has been shown that excessive local oxidative stress in the placenta, meaning excessive ROS production, inhibits nitric oxide (NO) activity, which, in turn, causes endothelial cell dysfunction, vasoconstriction, and reduced trophoblast invasion, leading to the development of preeclampsia (39). *OPRK1*, also known as KOR, is correlated with the production of ROS (40). Furthermore, in animal experiments, a κ-opioid receptor agonist has been found to stimulate NO production to attenuate the effects of hyperlipidemic damage on endothelial function (41). However, the specific mechanism of *OPRK1* in preeclampsia needs to be further investigated. *TPBG*, located at 6q14.1, encodes a leucine-rich transmembrane glycoprotein involved in cell adhesion. *TPBG* encodes a protein that is an oncofetal antigen targeting trophoblast cells. TPBG mRNA expression has been upregulated in circulating cells of preeclamptic women (42). Additionally, there are experiments confirming elevated *TPBG* expression in preeclamptic placentas, which may play a role in epithelial-mesenchymal transition during placental formation (43). As shown above, *INHBA* and *TPBG* have been relevant to the development of preeclampsia in experimental research, which is consistent with our findings obtained from the study. *OPRK1* has been correlated with oxidative stress in experimental research, which may be relevant to the development of preeclampsia. However, the exact association needs to be further verified. We constructed nomogram using *INHBA, OPRK1*, and *TPBG*. We applied ROC and DCA curves to validate the performance of the nomogram, which was also validated in the validation set, suggesting that the nomogram can indeed be used as a potential predictive model for preeclampsia. Thus, a nomogram may provide a simple and accurate method for predicting preeclampsia.

Subsequently, we analyzed the upstream regulation and found that *INHBA, OPRK1*, and *TPBG* were regulated by several transcription factors and other common mechanisms. Therefore, we performed the enrichment analysis of these transcription factors using cumulative recovery curves and found that the transcription factors REL, RELA, RELB, ZNF274, and SMARCC1 were the main regulators of *INHBA* and *TPBG*. Additionally, target miRNAs were predicted for *INHBA*, *OPRK1*, and *TPBG*, and the ceRNA network was constructed using Cytoscape. This network revealed regulatory mechanisms at the transcriptome level. Based on the TransmiR v2.0 database (44), which is a database used to get transcription factor (TF)-microRNA(miRNA) regulations based on *INHBA, OPRK1*, and *TPBG*, we found that RELA was associated with has-miR-548k and has-miR-1206. Therefore, we proposed that RELA-miR-548K/miR-1206-TPBG might be a potential RNA regulatory pathway controlling the progression of early preeclampsia disease. Further validation is still required to confirm our findings.

Many studies have proposed a therapeutic effect of curcumin in preeclampsia. At the cellular level, curcumin has been found to promote cell growth and migration in HTR8/SVneo trophoblast cells (a human placental model of first-trimester fetal cells), accompanied by the activation of Akt, anti-oxidative stress, and reduced methylation of DNA damage gene promoters, providing protection against placental diseases, such as preeclampsia (14). Curcumin has been found to play an anti-inflammatory role in human placental tissue (15). In animal models, curcumin (0.36 mg/kg) has reduced blood pressure and urinary protein levels and improves renal injury in a rat model of lipopolysaccharide (LPS)-induced preeclampsia-like phenotype (LPS of 0.5 ug/kg), and further placental tissue analysis has revealed that curcumin improved trophoblastic invasion defects and spiral artery remodeling and decrease nuclear factor kappa B (NF-κB), IL-6, and monocyte chemoattractant protein 1 (MCP-1) mRNA expression in serum and placenta (16). Additionally, curcumin has also been shown to inhibit placental inflammation through the upregulation of phosphorylated Akt, which effectively improves pregnancy outcomes in preeclampsia mice and increases live litter size, fetal weight, and placental weight (17). In this study, we selected the three key genes and RELA acting as targets for molecular docking with aspirin and curcumin, respectively. The binding energy between curcumin and TPBG-4cnc, OPRK1-4djh, and RELA-3qxy was less than −7.0 kcal/mol, which further suggested the effectiveness of curcumin for the treatment of preeclampsia. However, further validation is still needed to evaluate the safety.

Placenta, as a window of direct contact between a mother and fetus, is composed of different immune cells, with immune cells, stromal cells, and trophoblast cells forming an extensive network of cellular connections. Cellular immune imbalance is currently considered to be an influential factor in the development of preeclampsia. We performed an in-depth analysis of immune cell infiltration in placental tissue in preeclamptic and non-preeclamptic groups. Based on the CIBERSORT algorithm, we found that Tregs and resting mast cells were significantly higher in the preeclampsia group than in the non-preeclampsia group, whereas monocytes and neutrophils were significantly higher in the non-preeclampsia group than in the preeclampsia group. It is generally believed that Tregs have a strong suppressive activity and is an important role in attenuating destructive responses of the immune system during pregnancy and preventing autoimmune diseases (45).It is currently believed that the number of Tregs in the peripheral blood has the potential to be a biomarker for assessing the risk of preeclampsia, thus, facilitating the monitoring of patients with a high risk of pregnancy. However, these proposals are still in the research phase and require further systematic evaluation in more clinical trials. Tregs change throughout pregnancy, and reach a maximum in local and systemic Tregs expansion in mid-pregnancy (46), followed by a decrease in their proportion (47).A meta-analysis has shown that healthy pregnant women has a significantly higher number of Tregs in the peripheral blood compared to pregnant women with preeclampsia (standardized mean difference [SMD] = 1.46; 95% confidence interval [CI], 1.03–1.88; I^2^ = 92%; 30 studies). Healthy pregnant women has a higher number of Tregs in maternal meconium compared to pregnant women with preeclampsia, but there is no significant difference (SMD = 0.76; 95% CI, −0.13–1.65; I^2^ = 84%; 4 studies). It is speculated that this result may arise from the poor phenotype of the decidual tissue (48).The placental tissues collected in our article did not include the chorionic plate, let alone the decidua. The number of Tregs in the placental tissues was not consistent with the peripheral blood, which might arise from the poor phenotype or heterogeneity. Thus, the exact mechanism needs further investigation. Mast cells continuously examine the microenvironment to finely regulate immune responses (49).It has been indicated that abnormal activation of mast cells plays a crucial role in immune dysfunction (50).Mast cells have been correlated with vessel development (51).It has been found that mast cell expression is reduced in the placenta in early-onset preeclampsia, presumably because the reduction in mast cells causes abnormal spiral artery remodeling, which, in turn, affects placental villi development (52).There have been no studies on the correlation between resting mast cells and the development of preeclampsia. Preeclampsia pathogenesis is currently considered to be associated with the placental immune system. However, the specific impact of these immune cell differences on preeclampsia needs to be further investigated.

Preeclampsia is a multisystem disorder, characterized by abnormal placentation and maternal vascular dysfunction. Pathologic inflammation in early pregnancy leading to placental dysfunction and the subsequent entry of multiple placental factors into the maternal circulation, causing endothelial damage and clinical signs, are two widely accepted stages in preeclampsia development (26). Placental development is important for preeclampsia development. In this study, we explored the changes in inflammatory genes and related biological mechanisms at the maternal-fetal interface based on the differential expression of inflammatory genes in preeclampsia and non-preeclampsia groups. In recent years, several studies have found that placental RNA could be released into the maternal circulatory system during early gestation and that maternal plasma mRNA profiles correlate with placental gene expression profiles (53). A large number of placenta-specific genes have been identified in maternal plasma in preeclampsia, and measurement of circulating placental RNA (cpRNA) may provide an opportunity to predict maternal and fetal complications due to placental dysfunction in late pregnancy (54). Our study showed that placenta-specific inflammatory expression genes could be effective predictors for preeclampsia, and given the high risk and cost of obtaining placental tissue, we can attempt to further validate cpRNA in blood. Additionally, predicting drugs based on these measurements could provide a future for targeted treatment.

There are potential limitations to our study. First, as a retrospective study in a public database, there were limited data meeting the inclusion criteria; thus, further validation on a larger scale is expected. Second, our analysis was based on the genetic results of placental tissue, and the clinical application of this nomogram is limited given the high risk and cost of obtaining placental tissue. There are currently relevant experiments demonstrating some variation in blood, which we can further validate in blood. Additionally, since the data we analyzed came from public databases, further experimental studies are needed to validate the results of this study.

## Conclusion

In summary, we constructed machine learning models to identify three key genes that could be used as potential genetic biomarkers for the diagnosis and treatment of preeclampsia and established a prediction model. Subsequently, we further explored the potential transcription factors and miRNA-mRNA pathways upstream of the key genes and identified pathways that may help investigate the molecular mechanisms of preeclampsia and the development of therapeutic targets. With the use of established protein and ligand structure databases based on the key genes, we obtained predicted binding scores of drugs and prospects for their application. Finally, we explored the relevance of preeclampsia to the immune system. We hope that these findings will contribute to future in-depth studies to achieve the goal of improving the prognosis of patients with preeclampsia.

## Data Availability Statement

The datasets presented in this study can be found in online repositories. The names of the repository/repositories and accession number(s) can be found in the article/**Supplementary Material**.

## Ethics Statement

All data for this study were obtained from public databases and therefore did not require Institutional Review Board approval.

## Author Contributions

YZ conceived and designed the study and revised the content. YW made contributions to the data analysis and writing of the manuscript. BL Participated in writing. All authors contributed to the article and approved the submitted version.

## Funding

This work was supported by grants from the Science and Technology Projects of Liaoning Province (2021JH2/10300093).

## Conflict of Interest

The authors declare that the research was conducted in the absence of any commercial or financial relationships that could be construed as a potential conflict of interest.

## Publisher’s Note

All claims expressed in this article are solely those of the authors and do not necessarily represent those of their affiliated organizations, or those of the publisher, the editors and the reviewers. Any product that may be evaluated in this article, or claim that may be made by its manufacturer, is not guaranteed or endorsed by the publisher.
